# High-risk human papillomavirus (HPV) screening and detection in healthy patient saliva samples: a pilot study

**DOI:** 10.1186/1472-6831-11-28

**Published:** 2011-10-10

**Authors:** Deidre O Turner, Shelley J Williams-Cocks, Ryan Bullen, Jeremy Catmull, Jesse Falk, Daniel Martin, Jarom Mauer, Annabel E Barber, Robert C Wang, Shawn L Gerstenberger, Karl Kingsley

**Affiliations:** 1 University of Nevada, Las Vegas - School of Community Health Sciences, Department of Environmental and Occupational Health, Las Vegas, Nevada, USA; 2 University of Nevada, Reno - School of Medicine, Department of Surgery, Las Vegas, Nevada, USA; 3 University of Nevada, Las Vegas - School of Dental Medicine, Department of Biomedical Sciences, Las Vegas, Nevada, USA

## Abstract

**Background:**

The human papillomaviruses (HPV) are a large family of non-enveloped DNA viruses, mainly associated with cervical cancers. Recent epidemiologic evidence has suggested that HPV may be an independent risk factor for oropharyngeal cancers. Evidence now suggests HPV may modulate the malignancy process in some tobacco- and alcohol-induced oropharynx tumors, but might also be the primary oncogenic factor for inducing carcinogenesis among some non-smokers. More evidence, however, is needed regarding oral HPV prevalence among healthy adults to estimate risk. The goal of this study was to perform an HPV screening of normal healthy adults to assess oral HPV prevalence.

**Methods:**

Healthy adult patients at a US dental school were selected to participate in this pilot study. DNA was isolated from saliva samples and screened for high-risk HPV strains HPV16 and HPV18 and further processed using qPCR for quantification and to confirm analytical sensitivity and specificity.

**Results:**

Chi-square analysis revealed the patient sample was representative of the general clinic population with respect to gender, race and age (*p *< 0.05). Four patient samples were found to harbor HPV16 DNA, representing 2.6% of the total (n = 151). Three of the four HPV16-positive samples were from patients under 65 years of age and all four were female and Hispanic (non-White). No samples tested positive for HPV18.

**Conclusions:**

The successful recruitment and screening of healthy adult patients revealed HPV16, but not HPV18, was present in a small subset. These results provide new information about oral HPV status, which may help to contextualize results from other studies that demonstrate oral cancer rates have risen in the US among both females and minorities and in some geographic areas that are not solely explained by rates of tobacco and alcohol use. The results of this study may be of significant value to further our understanding of oral health and disease risk, as well as to help design future studies exploring the role of other factors that influence oral HPV exposure, as well as the short- and long-term consequences of oral HPV infection.

## Background

The human papillomavirus (HPV) has been implicated as the cause of virtually all cervical cancers worldwide [1-3]. These represent a large family of non-enveloped DNA viruses that may be found integrated into the host genome, non-integrated or episomal, or as a combination or mixture of these types in infected tissues [4-9]. HPV viruses infect many types of epithelial cells, with intraepithelial neoplasias accounting for the overwhelming majority of HPV-related cancers [5,10,11].

Recent epidemiologic evidence has suggested that HPV may also be an independent risk factor for oropharyngeal cancer, revealing HPV in three times as many pre-cancerous oral lesions, and almost five times as many oropharyngeal cancers compared with normal oral mucosa [12-14]. Of all HPV types, the high-risk strains HPV16, and to a lesser extent HPV18, are most commonly identified from oral biopsies [15-21]. Although the traditional risk factors for developing oropharyngeal cancer remain tobacco use and heavy alcohol consumption, other risk factors, such as HPV, may play significant roles in determining whether it develops and how quickly it may progress [14,19,22-28].

The comparatively low presence of high-risk HPV in normal tissues and much higher prevalence in pre-cancerous and cancerous oropharyngeal lesions may suggest that HPV preferentially infects already developing oropharyngeal cancers [12-14]. Although it is possible that the low prevalence in healthy individuals might be attributable to other factors, including improper specimen collection or assay sensitivity, it is also possible that HPV may function to modulate the malignancy process in developing or established oropharyngeal tumors, as has been observed in studies of HPV infection in other developing cancers [29-39]. For example, recent epidemiologic and case-control studies have demonstrated that patients with HPV-positive oropharyngeal tumors had significantly improved survival rates [12,40,41] and therapeutic response rates when compared with HPV-negative controls [42]. Several *in vitro *studies have recently investigated possible mechanisms that may account for these phenotypic changes in oropharyngeal cancers [25-27]. Evidence is now accumulating that HPV infection of some oropharyngeal cancers correlates with increased survival rates and better prognosis among some patients due to these changes in cellular responsiveness [40-45]. These studies highlight the need to understand not only the prevalence of oral HPV infection, but also the duration and persistence of such infections, due to their potential to affect oropharyngeal tumor progression.

It is likely that HPV may modulate the malignancy process in some tobacco- and alcohol-induced oropharyngeal cancers, but may also be the primary oncogenic factor for inducing carcinogenesis in a subset of patients without these traditional risk factors. Some evidence has demonstrated that non-tobacco and non-alcohol related oropharyngeal cancers were six times more likely to harbor HPV infections than case-matched controls [46]. To accurately evaluate risk, more estimates of oral HPV prevalence among healthy adults are needed. International studies have evaluated HPV prevalence in healthy adults using biopsy samples, revealing prevalence rates that ranged from 0 to 15% [20,47-51]. In addition, other studies have begun to report less invasive saliva and oral lavage-based testing methods to identify HPV among healthy adult saliva samples, revealing prevalence rates between 2.8 to 25% [15,52-60].

Some of these oral HPV screening studies were performed in the US on normal, healthy adults, in addition to oropharyngeal cancer patients [57,58,61]. These studies have reported much lower rates, between 1.3 and 7%, than in previous international studies [47,50,53]. Based upon this information, the goal of this project was to perform a screening for the most prevalent high-risk HPV strains, HPV16 and HPV18, in normal healthy adults. This pilot study was performed in Nevada, a state recently documented to have increasing rates of oropharyngeal cancer between 1997 and 2005 - despite declining rates of tobacco and alcohol use in the state, as well as declining rates of oropharyngeal cancer nationally [23,24]. The long-term goal is to provide more detailed information about high-risk oral HPV prevalence to allow for more robust estimates of oropharyngeal cancer risk.

## Methods

### Human Subjects

The protocol for this study titled "The Prevalence of Oral Human Papilloma Virus (HPV) in the University of Nevada, Las Vegas - School of Dental Medicine (UNLV-SDM) Clinic Population" was filed, amended, and approved by the UNLV Office of Research Integrity - Human Subjects (OPRS#1002-3361) on April 9, 2010. In brief, subjects in this convenience sample were recruited by members of the UNLV-SDM Clinic during their dental visit on one of 15 clinic dates. Informed Consent was required and was conducted onsite. Inclusion criteria: subjects had to be 18 years old or older and must agree to participate. Exclusion criteria: subjects younger than 18 years of age, subjects that declined to participate, and subjects with prior diagnosis of oropharyngeal cancer were excluded.

### Sample size

To determine an appropriate sample size, previous studies that screened for high-risk oral HPV in healthy adults were evaluated to determine the range of sample sizes, which varied greatly from 12 - 1,680 [47-62]. Previous research at UNLV and the UNLV-SDM clinic demonstrated low participation rates for invasive, blood-based screenings [63], but higher rates of participation using non-invasive biomonitoring and screening methods, including saliva collection (unpublished data). These studies had sample sizes ranging from 16 - 200. Based upon this combined information the maximum sample size was estimated to be 200.

### Saliva Collection Protocol

In brief, healthy adults who agreed to participate were given a small, sterile saliva collection container, a 50 mL sterile polypropylene tube from Fisher Scientific (Fair Lawn, NJ). Participants were then asked to chew on a small piece of paraffin wax for one minute and then to expectorate. Samples were stored on ice until transport to a biomedical laboratory for analysis. Each saliva sample was assigned a unique, randomly-generated number to prevent research bias. Demographic information regarding the sample was concurrently collected, which consisted of age, gender, and ethnicity only.

### Cell counting and DNA isolation

All samples were centrifuged for 10 minutes at 2,100 *g *(RCF) and the pellet washed with 1X phosphate-buffered saline (PBS) (HyClone: Logan, UT) and resuspended in 5 mL of 1X PBS. Cell number was determined using Trypan Blue (Fisher Scientific: Fair Lawn, NJ) using a Zeiss Axiovert 40 inverted microscope (Gottingen, Germany) and a hemacytometer (Fisher Scientific: Fair Lawn, NJ). To determine if any samples harbored the HPV virus, DNA was isolated from the saliva sample using a minimum of 3.5 × 10^5 ^cells and the GenomicPrep DNA isolation kit (Amersham Biosciences: Buckinghamshire, UK), using the procedure recommended by the manufacturer, as previously described [26,27,32]. DNA purity was calculated using ratio measurements of absorbance at 260 and 280 nm (A260/A280 ratio between 1.7 and 2.0).

### Polymerase chain reaction (PCR)

DNA from each sample was then used to perform PCR with the Fisher exACTGene complete PCR kit (Fisher Scientific: Fair Lawn, NJ) and a Mastercycler gradient thermocycler (Eppendorf: Hamburg, Germany) using the following primers for HPV16 [26,27], HPV18 [27,32], and glyceraldehyde- 3- phosphate dehydrogenase (GAPDH) [64], synthesized by SeqWright (Houston, TX):

HPV16 forward primer, ATGTTTCAGGACCCACAGGA;

HPV16 reverse primer, CCTCACGTCGCAGTAACTGT;

HPV18 forward primer, ATGGCGCGCTTTGAGGATCC;

HPV18 reverse primer, GCATGCGGTATACTGTCTCT;

GAPDH forward primer, ATCTTCCAGGAGCGAGATCC;

GAPDH reverse primer, ACCACTGACACGTTGGCAGT;

One μg of template DNA was used for each reaction. The initial denaturation step ran for three minutes at 94°C. A total of 30 amplification cycles were run, consisting of 30 second denaturation at 94°C, 60 seconds of annealing at 58°C, and 30 seconds of extension at 72°C. Final extension was run for five minutes at 72°C. The PCR reaction products were separated by gel electrophoresis using Reliant 4% NuSieve^® ^3:1 Plus Agarose gels (Lonza: Rockland, ME). Bands were visualized by UV illumination of ethidium-bromide-stained gels and captured using a Kodak Gel Logic 100 Imaging System and 1D Image Analysis Software (Eastman Kodak: Rochester, NY).

### Quantitative PCR (qPCR)

DNA samples were then processed using qPCR to provide more specific and sensitive quantification. Primers and probes were designed using Roche Universal Probe library (UPL) assay design software to amplify the region overlapping E6 and E7 gene sequence of HPV16 [GenBank: K02718] and the human β-actin housekeeping gene [GenBank: M10277]. All primers were purchased from Sigma-Aldrich (St. Louis, MO.) and probes purchased from Roche Applied Science (Indianapolis, IN.)

HPV16 E6/E7 forward primer 5'-CAACTGATCTCTACTGTTATGAGCAA-3', HPV16 E6/E7 reverse primer 5'-CCAGCTGGACCATCTATTTCA-3', HPV16 E6/E7 hydrolysis "Taqman" probe 5'-(fam)-AGGAGGAG-(dark quencher dye)-3' (UPL probe #63) was used to amplify the 73 base pair (bp) region between the 535 nucelotide (nt) position and 607 nt position. Human β-actin forward primer 5'-GTGGGGTCCTGTGGTGTG-3', human β-actin 5'-GAAGGGGACAGGCAGTGA-3', human β-actin hydrolysis "Taqman" probe 5'-(fam)-GGGAGCTG-(dark quencher dye)-3' (UPL probe #24) amplified the 61 bp region between 2642 nt position and 2702 nt position.

The real-time reaction mixture was prepared in a LightCycler^® ^480 multiwell Plate 96 containing 1× LightCycler^® ^480 Probes Master (Roche Applied Sciences), 1 μM of each respective primer set (forward and reverse), 0.2 μM of respective probe, and 2 μl of DNA template; in a 20 μl final reaction volume. The probes master mix contained reaction buffer, dNTP mix (including dUTP in place of dTTP), 3.2 mM MgCl_2_, and *Taq *DNA polymerase. The real-time PCR assay was performed on a LightCycler 480 system (Roche Applied Science) with the following cycle parameters: pre-incubation for initial enzyme activation at 95°C for 10 minutes, followed by 45 cycles of 95°C for 10 seconds (ramp rate 4.4°C/second), 60°C for 30 seconds (ramp rate 2.2°C/second) and 72°C for 1 second (ramp rate 4.4°C/second). Following amplification phase, a cooling step was performed at 40°C for 30 seconds (ramp rate of 2.2°C/second). Acquisition of the fluorescence signal was performed using Mono Hydrolysis Probe setting (465-510 nm) following the 72°C extension phase of each cycle. All samples were carried out in triplicate.

### Analytic sensitivity

The CaSki (American Type Culture Collection; Manassas, VA) cervical adenocarcinoma cell line was used to develop standard curves for both the HPV16 (600 copies/genome) and β-actin (2 copies/genome) genes. DNA extracted from CaSki cells was serially diluted ten-fold starting at 50 ng to 0.0005 ng [65]. This step allowed for relative quantification of the input DNA level and final quantity as the number of viral copies/genome/cell. Quantification was achieved using Cycle Threshold (C_T_) measured with the second derivative maximum method (LightCycler 480 Software version 1.5.0.39; Roche Applied Science). Saliva samples > 0.001 copy/genome were considered HPV positive. Specificity analysis was performed on qPCR assay against HPV18 and found to be 100% specific (data not shown).

### Statistical evaluation

Sensitivity and specificity were calculated as the proportion of true positives and true negatives (cutoff value > 0.001 copies/genome), respectively. Following the acquisition of saliva samples and HPV screening results, demographic information from each sample was compared with the overall demographic profile of the UNLV-SDM patient pool (N = 71, 051) using a chi-square (χ2) test, to determine if any characteristic (gender, race, age) was different than expected among the patients evaluated in this study (n = 151). A probability level of alpha (α) = 0.05 was used to determine statistical significance.

## Results

Samples of saliva were collected from 151 UNLV-SDM patients between June 2 and October 1, 2010. The patients from whom samples were collected and screened were not statistically different from the overall UNLV-SDM clinic population with respect to gender, race, or age (Table 1). More specifically, the total number of females and males in the sample was roughly equal (52.3% and 47.7%, respectively) and not significantly different than the overall clinic population (*p *= 0.589). There were slightly more White patients in the study sample (48.3%) than in the overall UNLV-SDM population (40.8%) (*p *= 0.133). In addition, there were slightly fewer 18 - 64 year olds in the sample (80.8%) than in the overall clinic (85.3%) (*p *= 0.354).

**Table 1:** Demographic analysis of study participants

Variables	UNLV-SDM	Study sample	Statistical analysis
*Gender*			
Female	n = 35,952 (50.6%)	n = 79 (52.3%)	χ2 = 0.119, d.f. = 1
Male	n = 35,099 (49.4%)	n = 72 (47.7%)	*p *= 0.589
*Race*			
White	n = 28,989 (40.8%)	n = 73 (48.3%)	χ2 = 1.621, d.f. = 1
Non-White	n = 42,062 (59.2%)	n = 78 (51.7%)	*p *= 0.133
*Age*			
18 - 64 years	n = 60,598 (85.3%)	n = 122 (80.8%)	χ2 = 1.056, d.f. = 1
65 +	n = 10,453 (14.7%)	n = 29 (19.2%)	*p *= 0.354

Analysis of saliva samples revealed cell counts varying between 0.8 - 2.4 × 10^6 ^cells/mL (Table 2). DNA was successfully isolated from all collected saliva samples, with average DNA concentrations ranging between 820 - 1047 ng/μL. Absorbance measurements and A260/A280 ratio analysis confirmed the purity of the DNA isolates, which averaged between 1.87 and 2.05.

**Table 2:** Cell count and DNA isolation

Cell count (cells/mL)	Average DNA concentration (ng/μL)	A260/A280	Samples (n)
0.8 - 1.2 × 10^6^	915	2.05	31
1.6- 1.9 × 10^6^	820	1.87	94
2.1 - 2.4 × 10^6^	1047	1.95	26

**Figure 1:**
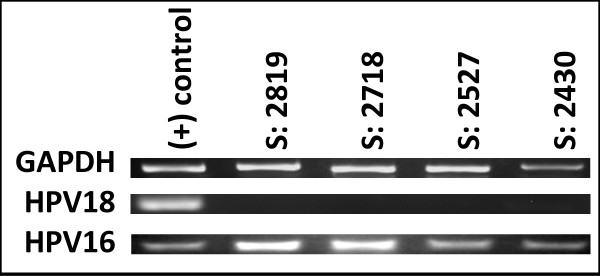
**Screening of patient samples for HPV**. PCR using DNA extracted from patient samples (n = 151) was screened using HPV16- and HPV18-specific primers, which revealed four samples harbored HPV16 (2819, 2718, 2527, and 2430). No samples were found to harbor HPV18. DNA extracted previously from cervical adenocarcinoma cell lines, CaSki and GH354, was used as HPV16 and HPV18 positive controls, respectively.

The extracted DNA was subsequently screened for the presence of HPV16 and HPV18 using PCR (Figure 1). From this screening, four patient samples were determined to be HPV- positive, which represented 2.6% of the total screened (n = 4/151). All four samples harbored HPV16 DNA and none were found to be positive for HPV18. Although a positive sample pool of only four patients does not allow for any broader inferences or conclusions, a preliminary descriptive analysis of demographic information revealed these four samples were from females, who were also minority (non-White, Hispanic) (Table 3). Three of the four samples were from patients between 18 - 64 years of age (2.0%), and one sample came from a patient over 65 years of age (0.7%).

**Table 3:** Analysis of HPVscreening

Variables	HPV16-positive	HPV18-positive	HPV-negative
*Gender*			
Female	n = 4 (2.6%)	n = 0	n = 75 (49.7%)
Male	n = 0	n = 0	n = 72 (47.7%)
*Race*			
White	n = 0	n = 0	n = 73 (48.3%)
Non-White	n = 4 (2.6%)	n = 0	n = 74 (49.0%)
*Age*			
18 - 64 years	n = 3 (2.0%)	n = 0	n = 119 (78.8%)
65 +	n = 1 (0.7%)	n = 0	n = 28 (18.5%)

**Figure 2:**
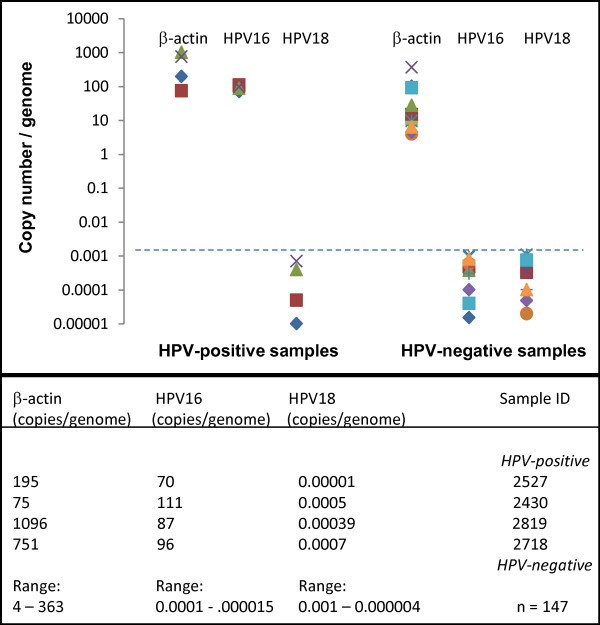
**Graphic analysis of qPCR HPV screening results**. Plotting of copy number/genome for housekeeping gene (β-actin) was similar from samples of HPV-positive (range: 75-1096) and HPV-negative samples (range: 4 - 363 copies/genome). Copy number/genome using qPCR was significantly above the cutoff value (> 0.1 copies/genome), confirming the HPV-positive samples did harbor HPV16 DNA (range: 70 - 111 copies/genome), but not HPV18. Values for HPV-negative samples were well beneath the cutoff value (range: 0.0003 - 0.000004 copies/genome), with the exception of three samples (2867, 2854, 2792) that were beneath the limit of detection.

DNA samples were then processed using quantitative qPCR to provide quantitative assessment, as well as measurement of sensitivity and specificity (Figure 2). Analysis of the range of copy number/genome for the housekeeping gene (β-actin) within HPV-negative (range: 4 - 363 copies/genome) and HPV-positive samples (range: 75-1096) were similar and well above the cutoff value (> 0.1 copies/genome). Analysis of qPCR results in copy number/genome for HPV revealed striking differences in copy numbers between HPV-negative (range: 0.0001 - 0.000004 copies/genome) and HPV-positive (range: 70 - 111), which were easily distinguished using the cutoff value (> 0.001 copies/genome). No false positives or false negatives were found, demonstrating sufficient sensitivity and specificity to ascertain the proportion of true positives (4/4 or 100%) and true negatives (147/151 or 100%).

## Discussion

The primary aim of this study was to perform an oral screening of normal healthy adults in Nevada for high-risk HPV. Previous attempts at this institution and within the state to obtain blood-based samples for other types of screenings, such as lead (Pb) levels, have been largely unsuccessful due to patient apprehension and fear of blood collection [63]. To avoid these specific problems, this study utilized non-invasively collected saliva to perform this analysis. These efforts ultimately allowed for the collection and screening of dozens of patient samples; a marked improvement in patient participation rates over other previous, but similar, attempts to collect biological specimens from the local population. The results of this current study provide new data from a previously unscreened patient population and geographic area to complement the growing corpus of information about oral HPV prevalence among healthy adults.

These data demonstrated a prevalence rate of high-risk oral HPV (2.6%) virtually the same as the most recent multinational studies of healthy, cancer-free adults (3.1% to 5%) [61,62]. Over the past few decades, international studies have evaluated HPV prevalence in healthy adults using biopsy samples, which reported widely variable prevalence rates that ranged from 0 - 15% [47-51]. However, other recently published reports screening for oral HPV infection among healthy adults using saliva and oral lavage testing reported overall prevalence rates that were also close to this range (1.3%, 2.8%, 7%) [52-59].

In addition, although the results of this study found oral HPV infection only among four patients, who were minority and female, the vast majority of female and minority patients in this study had no evidence of oral HPV infection. As an initial pilot study, these data suggest a more comprehensive and in-depth analysis of this population may be necessary, as recent epidemiologic studies have shown that rates of oropharyngeal cancer haven risen sharply among minority females in the US, despite overall declining rates [66]. More generally, rates of oropharyngeal cancer have risen among some minority subgroups [67], despite an overall decline among the general population in the US [23,24,68]. This may be explained, in part, by higher rates of tobacco and alcohol use, but may also be attributable to other factors including education, income, stress, diet, health literacy and exposure to oral infectious agents [22,25]. This pilot study provides preliminary information about oral HPV prevalence and the results suggest that further investigation may be warranted, particularly in light of the health disparities facing both females and minorities in Nevada and the US, in general [23,24].

This study also revealed the presence of the high-risk strain HPV16, but not HPV18. Although some reports have suggested that oral HPV infection may involve multiple HPV strains [15-21,69], this result is not dissimilar from other findings which suggest HPV16 may account for the overwhelming majority of HPV-positive oral samples [48,49,53,57,59]. It is likely that further expansion of this project to include larger samples will uncover additional HPV strains to provide more extensive estimates of oral HPV infection within this population.

This study had several limitations that should be considered. First, although the non-invasive nature of this study was sufficient for the recruitment and screening of a significant number of patients, the overall sample size was somewhat limited in comparison to the larger multinational studies previously mentioned [57,58,61]. The number of healthy adults screened for oral HPV in this pilot study, however, compares favorably with a number of other reports - with sample sizes ranging from 12 to 97 [20,47-53]. Second, detailed demographic and behavioral data were not designated as critical to the initial goals of this pilot study, however, the inclusion of smoking and tobacco use, as well as more detailed information about other behaviors, housing, education, income and other socioeconomic indicators, as well as sexual practices may provide additional insights in future investigations [55,58,59,70]. Finally, screening for additional high-risk HPV strains [71-75] and other oral infectious agents may be possible in future studies with more significant resources and personnel.

## Conclusions

This study successfully recruited patients and screened saliva samples for high-risk HPV, confirming HPV16, but not HPV18, was present in a small subset of the healthy adult patients in a US dental school clinic population. These patients were female and minority. Many health disparities face females and minorities in the US, and with some evidence suggesting that smoking and oropharyngeal cancer rates may be increasing in these population subgroups, the results of this study may be of significant value to other dental, medical, and health care professionals to further an understanding of oral health and disease risk.

## Abbreviations

(HPV): Human papillomavirus; (US): United States; (DNA): Deoxyribonucleic acid; (UNLV-SDM): University of Nevada, Las Vegas - School of Dental Medicine; (PCR): polymerase chain reaction; (GAPDH): glyceraldehyde-3- phosphate dehydrogenase; (qPCR): quantitative polymerase chain reaction; (bp): base pair; (nt): nucleotide; (RCF): Relative centrifugal force; (PBS): phosphate-buffered saline; (dNTP): Deoxyribonucleotide triphosphate; (dUTP): 2'-Deoxyuridine triphosphate; (dTTP): Deoxythymidine triphosphate.

## Competing interests

The authors declare that they have no competing interests.

## Authors' contributions

KK, SW, AB and RW conceived, monitored, and coordinated the experimental design. RB, JC, JF, DM, and JM were responsible for recruiting patients, informed consent, collecting samples, and some biomedical analysis. DT, KK, and SW carried out the DNA extractions, PCR, and qPCR analysis. KK, SW and DT were responsible for the data analysis, as well as the writing and editing of this manuscript. All authors have read and approved the final manuscript.

## Pre-publication history

The pre-publication history for this paper can be accessed here:

http://www.biomedcentral.com/1472-6831/11/28/prepub
